# Gastrointestinal Protein Hydrolysis Kinetics: Opportunities for Further Infant Formula Improvement

**DOI:** 10.3390/nu14071512

**Published:** 2022-04-05

**Authors:** Evan Abrahamse, Gabriël G. M. Thomassen, Ingrid B. Renes, Peter A. Wierenga, Kasper A. Hettinga

**Affiliations:** 1 Danone Nutricia Research, 3584 CT Utrecht, The Netherlands; gabriel.thomassen@danone.com (G.G.M.T.); ingrid.renes@danone.com (I.B.R.); 2 Food Quality and Design Group, Wageningen University and Research, 6708 WG Wageningen, The Netherlands; kasper.hettinga@wur.nl; 3 Department of Pediatrics, Emma Children’s Hospital, Amsterdam UMC—Location AMC, 1105 AZ Amsterdam, The Netherlands; 4 Laboratory of Food Chemistry, Wageningen University and Research, 6708 WG Wageningen, The Netherlands; peter.wierenga@wur.nl

**Keywords:** whey protein, caseins, cows’ milk, human breast milk

## Abstract

The postprandial plasma essential amino acid (AA) peak concentrations of infant formula (IF) are higher than those of human milk (HM) in infants. In addition, several HM proteins have been recovered intact in infant stool and appeared digestion resistant in vitro. We, therefore, hypothesized that gastrointestinal protein hydrolysis of IF is faster than HM and leads to accelerated absorbable digestion product release. HM and IF protein hydrolysis kinetics were compared in a two-step semi-dynamic in vitro infant digestion model, and the time course of degree of protein hydrolysis (DH), loss of intact protein, and release of free AA and peptides was evaluated. Gastric DH increase was similar for IF and HM, but the rate of intestinal DH increase was 1.6 times higher for IF than HM. Intact protein loss in IF was higher than HM from 120 min gastric phase until 60 min intestinal phase. Intestinal phase total digestion product (free AA + peptides <5 kDa) concentrations increased ~2.5 times faster in IF than HM. IF gastrointestinal protein hydrolysis and absorbable product release are faster than HM, possibly due to the presence of digestion-resistant proteins in HM. This might present an opportunity to further improve IF bringing it closer to HM.

## 1. Introduction

Human milk (HM) is the gold standard of infant nutrition and delivers nutrients to the infant to ensure the best possible growth and development [1]. When HM is not available, infant formula (IF) is an alternative. HM presents protein to the infant that is different in a.o. composition, processing, and matrix from IF, which can impact gastrointestinal digestion [2,3,4]. Since gastrointestinal protein digestion is a key determinant of systemic amino acid (AA) delivery rate and amount [5], efforts to support comparable protein digestion between HM and IF are warranted.

Protein is an essential nutrient as it is the only dietary source of (essential) amino acids (E)AAs. In the gastrointestinal tract, protein is digested to ultimately yield free AA, di-, and tripeptides, which can be absorbed by the small intestinal epithelium [6]. The kinetics of gastrointestinal protein digestion are determined by the rate of gastric emptying and protein hydrolysis by gastric, pancreatic, and intestinal brush border proteases and peptidases.

The postprandial (pp) plasma EAA peak concentration of humanized cow’s milk (HCM) protein-based IF was found to be 18% higher than that of iso-proteinaceous human milk (HM) in preterm infants [7]. This higher plasma peak could be due to differences in protein digestion and absorption kinetics in infants, as was shown for pp AA concentrations in adults [5]. Several studies have shown that the IF gastric emptying rate is similar to, or slower than, that of HM [8]. Together, this suggests that the release rate of absorbable protein digestion products upon hydrolysis of IF protein might be higher than that of HM protein.

The compositional humanization of cow’s milk (CM) protein in modern IF involves a change in casein to whey ratio (*c*/*w*) from 80:20 to 40:60 to mimic the ratio as found in mature HM [9]. However, casein and whey protein compositions differ between CM and HM. For example, the most abundant whey protein in CM is β-lactoglobulin, a protein that is absent in HM. In contrast, lactoferrin only constitutes 2% of the whey composition of CM, which in HM is the second most abundant whey protein [2]. As the protein hydrolysis rate depends on the protein type, these protein compositional differences may result in differences in the protein hydrolysis rate [10,11].

It has long been known that HM contains proteins that, in addition to EAA delivery, have functions in digestion and nutrient absorption, show anti-microbial activity, and are key in the development of the gastrointestinal and immune systems. Many of these functional proteins depend on their intact structure to exert their function in the gastrointestinal tract [9]. As such, some are relatively resistant to digestion, for example, lactoferrin which can be retrieved intact in low amounts (6–10% of intake) in the stool of breastfed infants [12]. In addition, human milk contains inhibitors of gastrointestinal proteases [13], possibly to protect the functional proteins from breakdown, which may also affect overall protein hydrolysis kinetics.

Additionally, milk protein processing and product matrix, which are very different between HM and IF have both shown contradictory effects on protein hydrolysis rate and pp AA concentrations [4,14]. Still, higher plasma EAA concentrations have been observed after IF ingestion [7]. We hypothesize that the release rate of absorbable products of protein digestion of IF protein is higher than HM. The aim of this study is to compare the time course of protein hydrolysis of HM and IF in a two-step semi-dynamic in vitro model of the infant gastrointestinal tract (SIM). The in vitro model does not contain brush border enzymes that are responsible for releasing absorbable products from intermediate peptides. Therefore, the time course of release of not only free AA (FAA), di- and tripeptides but also intermediate digestion products (medium molecular weight peptides (MMW, <5 kDa)) are evaluated. Additionally, the time course of the degree of protein hydrolysis (DH) and loss of intact protein are assessed to obtain insights into the potential underlying and driving mechanisms.

## 2. Materials and Methods

### 2.1. Materials

The composition of simulated infantile digestive fluids was as follows. Simulated saliva fluid (SSF) consisted of: 0.6 g/L α-amylase (Aspergillus oryzae, Sigma A9857, 150 units/mg protein [15], Sigma-Aldrich Chemie N.V., Zwijndrecht, the Netherlands), 6.2 g/L NaCl, 2.2 g/L KCl, 0.3 g/L CaCl_2_∙2H_2_O and 1.2 g/L NaHCO_3_ in distilled water, adjusted to pH 6.3. Simulated gastric fluid (SGF) consisted of: 125 mg/L lipase (Rhizopus oryzae, Amano DF 15, 177 FIP units/mg [16], Amano Enzyme Europe Limited, Oxfordshire, United Kingdom), 50 mg/L pepsin (Porcine, Sigma P7012, 2500 units/mg protein [17]), 3.1 g/L NaCl, 1.1 g/L KCl, 0.15 g/L CaCl_2_∙2H_2_O, 0.82 g/L Na-acetate, in distilled water, adjusted to pH 5.8. Simulated intestinal fluid (SIF) consisted of: 5.0 g/L bile extract (Porcine, Sigma B8631), supernatant of centrifuged (12,000× *g* for 20 min at 4 °C) pancreatin (Porcine, 4×USP unit activity [16], Sigma P1750) 12.5 g/L, 2.5 g/L NaCl, 0.3 g/L KCl, 0.15 g/L CaCl_2_∙2H_2_O adjusted to pH 7.0. Simulated digestive fluids were prepared freshly. The protease inhibitor used was Bowman-Birk inhibitor (BBI) (Sigma T9777), all other chemicals were of analytical grade and obtained from Sigma or Merck (Merck Life Science NV, Amsterdam, The Netherlands).

### 2.2. Sample Description

HM was obtained from 8 donors in The Netherlands from March 2015 to February 2018, after signing written informed consent. Donors indicated having surplus milk that was not needed to feed their infants. In total, 17 mature HM donations were made on average on day 157 after term delivery (range 60–251, median 167). Collection took place at least one hour after nursing the infant. Collected milk was stored directly at −80 °C. On the day before testing in late 2019, donated milk was thawed by placing at 4 °C, and subsequently pooled. Infant formula (IF) suitable for infants up to 6 months of age was bought in a local supermarket. The IF reconstitution rate (11.85% (*w*/*v*)) was different from recommendations on the pack (13.60% (*w*/*v*)) to match the HM pool protein equivalent content. Infant milk protein equivalent (P_eq_) was defined to consist of “true protein” as defined by Lonnerdal [9], and FAA, and was calculated using Equation (1):

(1) $$P_{\mathrm{eq}}\left(0\right)\;\left[g/L\right]=\left(\left[N\right]-\left[\mathrm{NPN}\right]\right)\times 6.25+\left[\mathrm{AA}\right]$$

where [N] is the concentration of nitrogen present in the infant milk as quantified using the Dumas method, [NPN] is the concentration of non-protein nitrogen (NPN, N soluble in 12% (*w*/*v*) trichloroacetic acid), 6.25 is the protein conversion factor, and [AA] is the concentration of free AA in infant milk quantified by UPLC as described below. FAA were included in the calculation of protein equivalent because they are excluded from the “true protein” concentration, as they are soluble in 12% trichloroacetic acid, while they are a source of AA to the infant. P_eq_(0), as we define it, does not include all LMW and MMW peptides present in HM and IF because it is unknown whether these peptides are all soluble in 12% trichloroacetic acid (i.e., it is unknown if they all contribute to NPN). Infant milk nutritional composition is given in Table 1.

### 2.3. Semi Dynamic In Vitro Simulation of Infant Gastrointestinal Tract (SIM)

**Figure 1:**
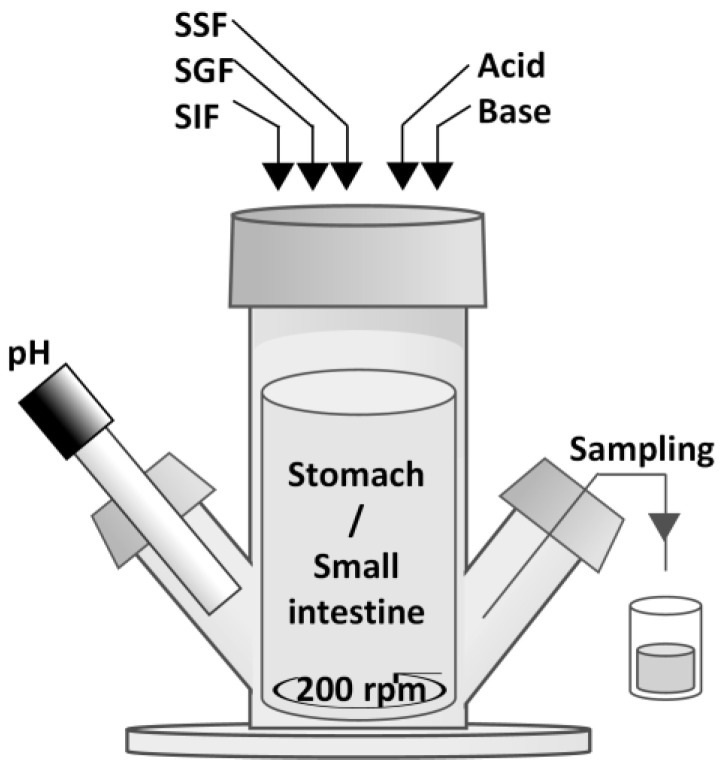
Schematic representation of SIM in vitro model of the infant gastrointestinal tract. SSF = simulated saliva fluid, SGF = simulated gastric fluid, SIF = simulated intestinal fluid.

**Figure 2:**
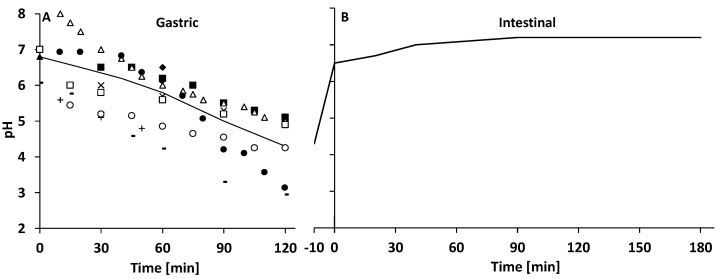
SIM pH curves. (**A**) Pre-set gastric pH curve (straight line) and in vivo reference values from: ■ [21], ♦ [22], ▲ [23], ✕ [24], **○** [25], ▪ [26], + [27], **△** [28], ● [29], and **☐** [30]. (**B**) Pre-set intestinal pH curve based on values used by Blanquet et al. in TIM-1 [31]. Pre-set gastric pH curve is polynomial (3rd order) trendline of mean in vivo pH per timepoint.

HM and IF were digested in vitro using the SIM. The SIM is based on a computer-controlled parallel fed-batch system by Dasgip equipped with 100 mL bioreactors (Eppendorf, Dasgip Mini Spinner Type DS0100B, Eppendorf Nederland BV, Nijmegen, the Netherlands) (Figure 1) [18,19]. Ratios of milk to simulated digestive fluid were chosen to simulate the ingestion of a 200 mL meal by a 0–6-month-old infant. The start volume of the bioreactors was 35 mL milk and all volumes were adjusted proportionally to this volume. Milk to simulated digestive fluid ratios and enzyme activity at the end of the digestion phases resembled recommendations for the static in vitro infant digestion model from INFOGEST [20]. In contrast to the INFOGEST recommendations, we did not use gastric lipase from rabbit, but a fungal lipase as described previously [18,19]. Prior to the digestion experiment, bioreactors were filled with 37 mL of infant milk. Bioreactor temperature was maintained at 37 °C using a water bath. After the temperature of the milk reached 37 °C, a 2.0 mL sample was taken from the bioreactors and the gastric phase of 120 min was started by a single shot of SSF and SGF, followed by continuous SGF addition. During the gastric phase, the pH was gradually lowered following a set curve based on in vivo observations by the addition of 1 M HCl to closely mimic the dynamic postprandial infant gastric pH (Figure 2A). After the gastric phase, the pH was increased to 6.5 in 10 min by the addition of 1 M NaHCO_3_. The subsequent intestinal phase of 180 min was started by a single shot of SIF followed by continuous SIF addition. During the intestinal phase, the pH was gradually increased to 7.2 at 180 min by addition of 1 M NaHCO_3_ (Figure 2B). Digesta samples (2.0 mL) were taken at 10, 30, 60, 90, and 120 min of gastric phase and 2, 6, 10, 20, 30, 60, 120, and 180 min of intestinal phase. Gastric and intestinal digesta were visually homogeneous and no large lumps were present that could hamper pipetting, which indicates digesta samples were representative. Digesta samples were directly diluted 1:1 with sample buffer (0.1 M phosphate buffer) and snap-frozen in liquid nitrogen. Sample buffer pH and content were chosen to inhibit enzymatic activity during storage. Gastric sample buffer was pH 7, intestinal sample buffer was pH 5.5, and contained 0.58 g/L BBI. Blank runs, using phosphate-buffered saline to replace milk, were performed to determine the contribution of the added simulated digestive fluids to the total concentrations of amino groups, peptides, and AA.

### 2.4. O-Phthalaldehyde Method (OPA)

To determine the degree of hydrolysis (DH), free amino groups were quantified using the OPA method. The OPA reagent was prepared as described previously [32]. Samples were diluted to 5 g protein /L in a 20 g SDS /L solution, stirred for 20 min, and stored at 4 °C overnight. The samples were then diluted to 2 g protein /L with Millipore water. Aliquots (5 μL) were added to 300 μL of the OPA reagent solution and equilibrated for 10 min. The presence of alkyl-iso-indols formed by the reaction of free amino groups with OPA was measured by the absorbance at 340 nm. To calculate the concentration of free NH_2_ groups, a calibration curve was measured using leucine as a reference compound. The total concentration of amino groups of HM and IF was determined by OPA after hydrolysis in 6 M HCl at 110 °C for 22 h. The degree of hydrolysis (DH) at a time point (t = y) was calculated using Equation (2):

(2) $$\mathrm{DH}\left(t\right)\;\left[\%\right]=\frac{{\left[{\mathrm{NH}}_{2}\right]}_{t}\times \mathrm{df}-{\left[{\mathrm{NH}}_{2}\right]}_{0}}{{\left[{\mathrm{NH}}_{2}\right]}_{\mathrm{AH}}-{\left[{\mathrm{NH}}_{2}\right]}_{0}}\times 100$$

where ${\left[{\mathrm{NH}}_{2}\right]}_{t}$ is the concentration of free amino groups present in the t = y digesta sample, df = dilution factor during digestion, ${\left[{\mathrm{NH}}_{2}\right]}_{0}$ is the concentration of free amino groups present in the t = 0 sample, and ${\left[{\mathrm{NH}}_{2}\right]}_{\mathrm{AH}}$ is the concentration of free amino groups present in the acid-hydrolyzed t = 0 sample of the infant milk. The total infant milk peptide bond concentration is calculated by subtracting ${\left[{\mathrm{NH}}_{2}\right]}_{0}$ from ${\left[{\mathrm{NH}}_{2}\right]}_{\mathrm{AH}}.$ The DH increase per phase (gastric or intestinal) is calculated by subtracting the corresponding DH(0) from DH(t).

### 2.5. Sodium Dodecyl Sulfate-Polyacrylamide Gel Electrophoresis (SDS-PAGE)

Intact protein in HM, IF, and digesta samples was analyzed by reducing SDS-PAGE using NuPAGE 4–12% Bis-Tris Midi protein precast gels (WG1402A Invitrogen, Thermo Fisher Scientific, Landsmeer, the Netherlands). Samples were diluted to a standardized protein concentration of 0.23 g/L using demineralized water. Subsequently, 10 µL of lithium dodecyl sulfate sample buffer (NuPAGE™ lithium dodecyl sulfate sample buffer (4×), NP0007 Invitrogen, Thermo Fisher Scientific) and 4 µL of sample reducing agent containing 500 mM of dithiothreitol (NuPAGE™ sample reducing agent (10×), NP0009, Invitrogen, Thermo Fisher Scientific) were added to 26 µL of diluted sample. Subsequently, samples were heated for 10 min at 70 °C. Each lane was loaded with 20 µL of heated sample, i.e., 6.6 µg of protein. Gels were run using SDS containing running buffer (XT MES running buffer, 1610789, Bio-Rad, Veenendaal, the Netherlands). Gels were fixed in 40% (*v*/*v*) methanol, 10% (*v*/*v*) acetic acid, rinsed, and stained with SimplyBlue SafeStain (LC6060 Invitrogen, Thermo Fisher Scientific). PageRuler™ Plus prestained protein ladder (PI26620, Fischer Scientific) was used as a molecular-weight marker. Gels were imaged using a BIO-RAD Gel Doc XR imager and band intensity was quantified using Quantity One software. The relationship between protein quantity and band intensity was calibrated using five concentrations of a whey protein concentrate in demineralized water, showing linearity from 1 to 5 ug total protein per lane. The proportion of total intact milk protein remaining at a time point (t = y) during SIM digestion was calculated by expressing the sum of the intensity of all bands at t = y (corresponding to bands present at t = 0) as a proportion of the intensity of all bands at t = 0 (% of P_i_(0)). The same procedure was followed for individual proteins (x) present as single bands at t = 0 and t = y and is expressed as (% of P_xi_(0)).

### 2.6. Ultra High-Performance Liquid Chromatography-Fluorescence (UPLC-FLR)

Quantification of free AA using UPLC-FLR was performed as described earlier [18]. Briefly, infant milk and digesta samples were prepared for elution by precipitation of proteins and large peptides with 3.5% (*w*/*v*) HClO_4_ and filtration. The concentration of each AA in the sample was determined by UPLC using a pre-column derivatization with OPA and fluorometric detection. AA quantity in digesta samples is expressed as the weight percentage of respective infant milk protein equivalent (% of P_eq_(0), Table 1). Cysteine, methionine, and proline could not be quantified under the conditions used.

### 2.7. High-Performance Size Exclusion Chromatography (HP-SEC)

Peptides were separated by size and then quantified using size exclusion chromatography (SEC) as described earlier [18]. The HPLC system (Shimadzu, ’s-Hertogenbosch, the Netherlands) was equipped with a Superdex Peptide 10/300 column (17-5176-01 GE Healthcare, München, Germany) with a 10 kDa HMW cut off. Samples were centrifugated for 5 min (12,000× *g*), after which 20 µL of supernatant was injected onto the column. The eluent was 25% (*v*/*v*) acetonitrile, 16% (*v*/*v*) trifluoracetic acid and detection was performed by absorption at 200 nm. The relationship between elution time and molecular weight was established using ten standards; Cytochrome C from bovine heart (12,327 Da), Aprotinin from bovine lung (6500 Da), Adrenocorticotropic hormone from porcine pituitary (4567 Da), Insulin A-chain oxidized ammonium salt from bovine pancreas (2532 Da), Angiotensinogen 1–14 Renin substrate porcine (1759 Da), Bradykinin acetate salt (1060 Da), Bradykinin Fragment 1–7 (757 Da), Bradykinin Fragment 1–5 (573 Da), Ala-Ala-Ala-Ala-Ala (373 Da), and Gly-Leu (188 Da). The correlation coefficient of the linear fit between elution time between 9.2 and 19.2 min and 10 log molecular weight was 0.980. The chromatogram of a tryptophane standard showed that this amino acid started to elute at 23.0 min. Therefore, chromatograms were integrated from 11.4 min (5 kDa) to 23.0 min to exclude free amino acids that absorb at 200 nm. Peptide quantity was calculated using estimation of peptide amino acid composition and peptide molar extinction coefficients using the methodology and reported extinction coefficients by Kuipers and Gruppen [33]. The average extinction coefficient of the peptides in the integrated area $\bar{\varepsilon \left(x\right)}$ was calculated using Equation (3), adapted from [34],

(3) $$\bar{\varepsilon \left(x\right)}=\varepsilon_{b}\times \left(\frac{\mathrm{Mw}\left(\bar{x}\right)}{\mathrm{Mw}\left(\bar{\mathrm{AA}}\right)}-1\right)+\varepsilon \bar{\left(\mathrm{AA}\right)}\times \frac{\mathrm{Mw}\left(x\right)}{\mathrm{Mw}\bar{\left(\mathrm{AA}\right)}}$$

where $\varepsilon_{b}$ is the extinction coefficient of a peptide bond, which equals 923 M^−1^cm^−1^, $\mathrm{Mw}\left(\bar{x}\right)$ is the mean peptide molecular weight in the integrated area, $\mathrm{Mw}\bar{\left(\mathrm{AA}\right)}$ is the average amino acid molecular weight in the mean peptide,$\;\varepsilon \bar{\left(\mathrm{AA}\right)}$ is the weighted average extinction coefficient of the amino acids in the mean peptide. Peptides were clustered in two fractions, LMW <0.5 kDa and MMW from 0.5–5 kDa. Peptides <0.5 kDa were considered di- and tri- peptides. LMW and MMW peptide quantity in digesta samples is expressed as weight percentage of respective infant milk protein equivalent (% of P_eq_(0)).

### 2.8. Data Analysis

Data obtained from HM and IF were corrected for the level found in blank runs where appropriate. Data are shown as the mean ± standard error of the mean (n = 3) unless specified otherwise. The concentration of total digestion products (TP) was calculated as the sum of FAA, LMW, and MMW concentrations. Parameters (DH, FAA, LMW, and TP) measured in the intestinal phase were fitted using first-order reaction kinetics using Equation (4):

(4) $$z\left(t\right)=m\times (1-e^{-\mathrm{kt}})+b$$

where z(t) is the value of the parameter at time t, m is the maximal increase in value that can be reached, k is the rate constant, and b is the value at 120 min gastric phase. The solver function in Microsoft Excel was used to calculate the values for k, m, and b that resulted in the lowest sum of squares of residuals. Results of k, m, and b are reported with suffix to denote the respective parameter (DH, FAA, LMW, and TP). The statistical significance of differences was analyzed by the Kruskal–Wallis test in SPSS 19. Differences with a *p*-value below 0.05 were considered statistically significant. 

## 3. Results

### 3.1. Characterization of Human Milk and Infant Formula

Nitrogen (N) and non-protein N (NPN) concentrations were, respectively, ~0.06 g/L and ~0.12 g/L higher in HM than in IF, leading to a lower true protein concentration in HM than IF (Table 1). In contrast, HM total FAA concentration, as determined using UPLC-FLR, was ~14 times that of IF. Approximately 70% (*w*/*w*) of HM FAA consisted of glutamine and glutamic acid. The protein equivalent (P_eq_(0), sum of true protein and FAAs) was similar for HM and IF and consisted of 96.26 and 99.79% of true protein, respectively, with the remainder being FAA. Low molecular weight peptide (LMW, <0.5 kDa) concentrations in HM, as determined using HPSEC, were ~3 times higher in HM than in IF (9.27 ± 1.65 and 2.95 ± 1.05% of P_eq_(0), respectively). However, medium molecular weight peptide (MMW, 0.5–5 kDa) concentrations were similar (11.40 ± 6.32 and 8.29 ± 1.60% of P_eq_(0) for HM and IF, respectively) (see Section 3.2.1 for more details). The sum of FAA, LMW, and MMW peptide concentrations, (during digestion referred to as total digestion products (TP)), was similar in HM and IF. If it is assumed that LMW and MMW peptides, as determined using HPSEC, are not intact proteins and are not soluble in TCA (thus included in “true protein”), then this would mean that the intact protein concentration is 75.59 and 88.55% of P_eq_(0) for HM and IF, respectively. Similar total peptide bond concentrations were found in HM and IF, being 85.2 ± 4.9 and 88.8 ± 2.6 mM, respectively, as determined using OPA. Protein composition analysis using SDS-PAGE, as shown in Table 2, revealed that the six most abundant proteins in HM together constitute ~96% of P_i_(0), in order of abundance: α-lactalbumin, lactoferrin, β-casein, serum albumin, free secretory component (SC), and κ-casein. In IF, six proteins were detected (adding up to 100%); in order of abundance: β-lactoglobulin, β-casein, κ-casein, α-lactalbumin, α-casein, and serum albumin.

### 3.2. Gastrointestinal Protein Hydrolysis in SIM

#### 3.2.1. Gastric Digestion

**Figure 3:**
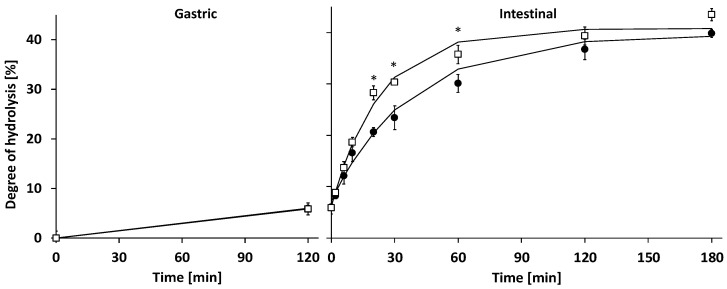
Protein degree of hydrolysis in gastric and intestinal digesta in time. (●) Human milk (HM), (**☐**) infant formula (IF); lines in the intestinal phase are fitted data using first-order reaction kinetics; mean ± SEM (n = 3). * Significant difference (*p* < 0.05).

**Figure 4:**
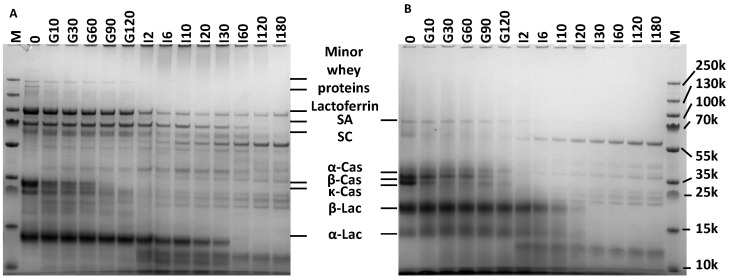
Typical SDS-PAGE gels loaded with undigested milk and digesta samples. (**A**) Human milk (HM), (**B**) infant formula (IF). Lanes are marked with type of sample with prefix indicating the digestion phase (G = gastric, I = intestinal) and number reflecting time in min. M = molecular weight marker. SA = serum albumin, SC = secretory component (of immunoglobulins), α-Cas = alpha-casein, β-Cas = beta-casein, κ-Cas = kappa-casein, β-Lac = beta-lactoglobulin; α-Lac = alpha-lactalbumin. Annotation based on [2,11].

**Figure 5:**
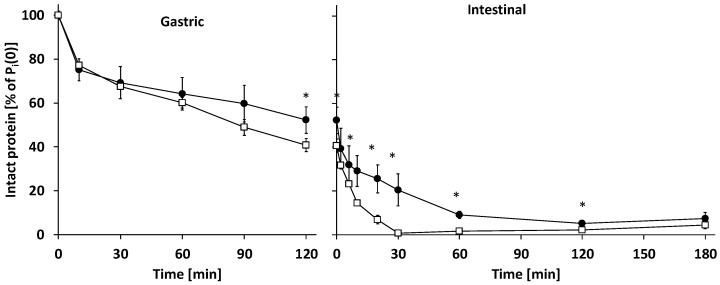
Total intact protein hydrolysis in gastric and intestinal digesta in time, as determined by SDS-PAGE and densiometric analysis of bands. (●) Human milk (HM), (**☐**) infant formula (IF); total intact protein expressed as % of total intact milk protein amount at t = 0 (P_i_(0)). Mean ± SEM (n = 3). * Significant difference (*p* < 0.05).

**Figure 6:**
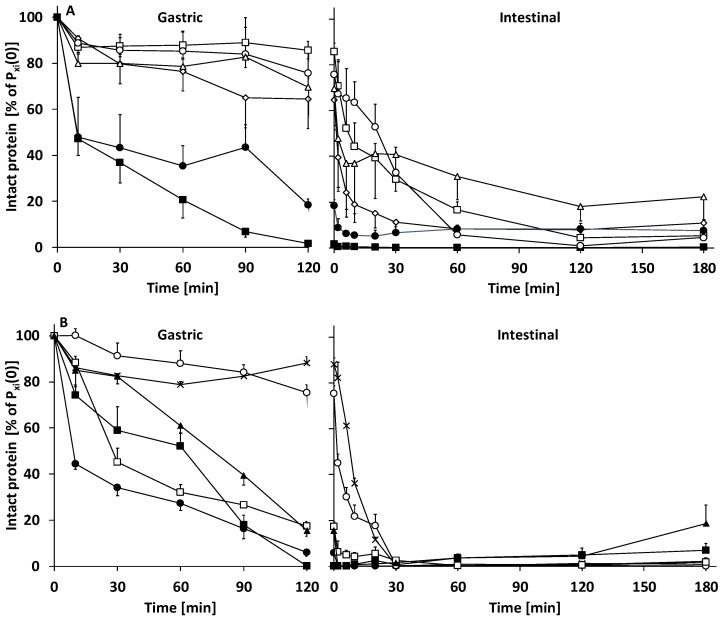
Individual intact protein hydrolysis of the six most abundant proteins in gastric and intestinal digesta in time, as determined by SDS-PAGE and densiometric analysis of bands. (**A**) Human milk (HM), (**B**) infant formula (IF); (◇) lactoferrin, (☐) serum albumin, (○) α-lactalbumin, (△) secretory component (of immunoglobulins), (✕) β-lactoglobulin, (▲) α-casein, (■) β-casein, and (●) κ-casein. Intact protein expressed as % of respective intact milk protein amount at t = 0 (P_xi_(0)). Mean + or − SEM (n = 3).

**Figure 7:**
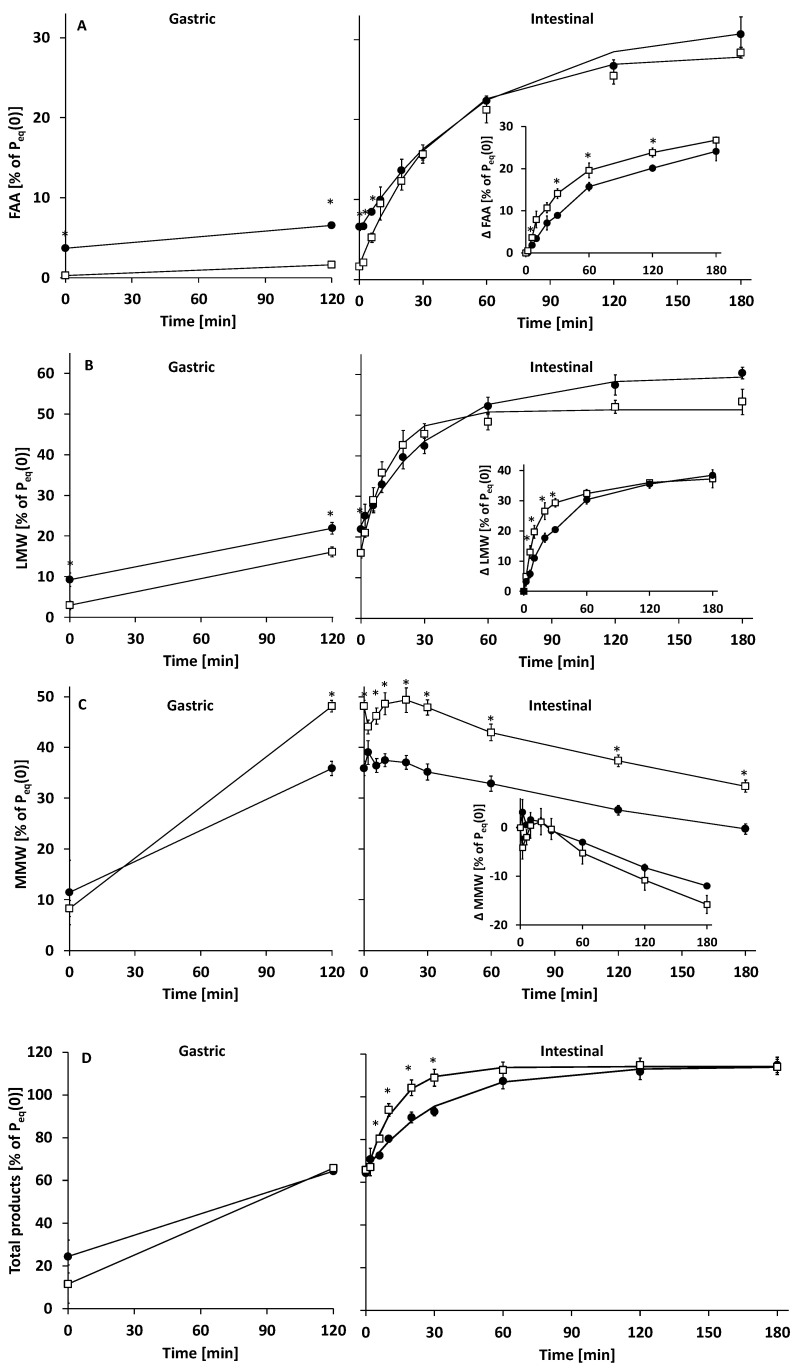
Protein digestion products of human milk (HM, (●) and infant formula (IF, **☐**) in time, expressed as weight percentage of protein equivalent in the respective infant milk (% of P_eq_(0)). (**A**) free amino acids (FAA), (**B**) Low molecular weight peptides (LMW, <0.5 kDa), (**C**) medium molecular weight peptides (MMW, 0.5–5 kDa) and (**D**) total protein digestion products (TP, sum of FAA, LMW, and MMW). As determined by UPLC (**A**), as determined by HP-SEC (**B**,**C**). Lines in (**A**–**D**) intestinal phase are fitted data using first order reaction kinetics. Inserts in (**A**–**C**) show respective protein digestion products time course vs. t = 0 intestinal phase; mean ± SEM (n = 3). * Significant difference (*p* < 0.05).

In the gastric phase (0–120 min), milk protein degree of hydrolysis (DH) increased to ~6.0% at 120 min for both IF and HM (Figure 3). Gastric protein hydrolysis caused extensive intact protein loss, which at 120 min was higher for IF than HM: 40.7 ± 3.0 and 52.3 ± 6.0% of P_i_(0) remained intact, respectively (Figure 4 and Figure 5). Intact protein loss at 120 min for all caseins was high in both HM and IF: up to 20% remained intact (Figure 6). Intact whey protein was digested considerably less than casein; in both HM and IF, whey proteins were still maximally up to 65% intact at 120 min, except for intact serum albumin in IF, of which only 18% remained intact (Figure 6B). Gastric protein hydrolysis was accompanied by less than 3% of P_eq_(0) FAA release for both HM and IF (Figure 7A). The increase in LMW peptides was similar in HM and IF: 12.6 ± 3.08 and 13.1 ± 2.27% of P_eq_(0), respectively (Figure 7B). Substantially more MMW peptides were released from IF than HM: 39.9 ± 2.75 vs. 24.4 ± 7.75% of P_eq_(0), respectively (Figure 7C). The concentration of total digestion products (sum of FAA, LMW, and MMW) at the end of the gastric phase was similar at ~60% of P_eq_(0), although the increase was larger in IF due to a lower concentration at t = 0 (Figure 7D).

#### 3.2.2. Intestinal Digestion

Intestinal DH increase was faster for IF than HM; k_DH_ of IF was 1.6 times higher than of HM (Table 3) and IF DH from 20–60 min was also higher than HM DH (Figure 3). However, from 120 min onwards, DH was similar again for IF and HM. Additionally, m_DH_ was also similar for IF and HM, indicating that the same DH plateau was being reached, albeit at a slower rate for HM. Intestinal total intact protein loss of IF was considerably faster than that of HM. After 30 min, IF intact protein loss was complete (intact protein = 0% P_i_(0)), while HM intestinal digesta still contained 20.4 ± 6.4% of P_i_(0) intact protein at 30 min (Figure 5). At this digestion time point, ~11% lactoferrin, ~30% α-lactalbumin and serum albumin, and ~40% free secretory component in HM were still intact (Figure 6A). SDS-PAGE of intestinal digesta samples showed several bands (a.o. at 25, 35, and 60 kDa) increasing in density over time (Figure 4). These bands may represent pancreatic enzymes present in SIF (i.e., trypsin, chymotrypsin/elastase, and triglyceride lipase/α-amylase, respectively, based on their molecular weight) or breakdown products of larger milk proteins. Intestinal protein hydrolysis was accompanied by a similar FAA release rate (k_FAA_) for both HM and IF (Table 3). As a consequence of the higher FAA concentration in undigested HM, FAA concentrations up to 6 min intestinal phase and b_FAA_ were both higher for HM than in IF (Figure 7A, Table 3). However, the concentration of released FAA was higher at several timepoints in IF than in HM (Figure 7A insert). Undigested HM contained higher LMW peptide concentrations than IF, and gastric release of LMW peptides was similar, resulting in higher intestinal phase starting LMW concentration (b_LMW_) for HM (Figure 7B, Table 3). IF intestinal LMW release rate was 2.6 times that of HM (k_LMW_ in Table 3). The concentration of released LMW was also higher from 6–30 min in IF than HM (Figure 7B insert). During the intestinal phase, IF digesta MMW concentration was consistently higher than that of HM digesta (Figure 7C). However, the MMW concentration decrease in time was similar for both HM and IF (Figure 7C insert). The biggest difference was at 30 min, where the IF digesta MMW concentration was 1.4 times that of HM. The concentration of TP (sum of FAA, LMW, and MMW) in the intestinal phase increased ~2.5 times faster in IF than in HM (k_TP_ in Table 3). The concentration of released TP was also higher from 6–30 min in IF than in HM (Figure 7D). The end concentration of TP in the intestinal phase was similar at ~114% of P_eq_(0) (>100% reflecting the contribution of LMW and MMW peptides present in undigested milk which were not included in P_eq_(0), and the addition of water molecules in the hydrolysis process, which contributes ~6.6% P_eq_(0) at the end of digestion).

## 4. Discussion

In the current study, we compared in vitro the time course of protein hydrolysis of HM with that of an HCM based IF, using gastrointestinal conditions mimicking those in infants. We hypothesized that the absorbable digestion product release rate of IF would be higher than that of HM. This hypothesis was based on the observation by Moro et al., that postprandial plasma EAA peak concentrations of IF were higher than those of HM [7], and the observations that several HM proteins were more hydrolysis resistant than IF proteins [2,9,12]. In line with our hypothesis, it was observed that the concentration of TP (sum of FAA, LMW, and MMW) in the intestinal phase increased ~2.5 times faster in IF than in HM (Figure 7D, Table 3), mostly due to the differences in MMW concentrations: The intestinal MMW peptide peak concentration was 1.4 times higher for IF than HM (Figure 7C). Additionally, the rate of intestinal DH increase was 1.6 times higher for IF than in HM (Table 3). Intact protein loss was higher in IF than in HM from 120 min gastric phase until 60 min intestinal phase (Figure 5).

In contrast to our observations that IF has a higher digestion product release rate than HM, Maathuis et al. reported a lower protein digestion rate from HCM protein-based IF than HM [35]. In the reported study, the digestion rate was assessed as the accumulation of N in intestinal dialysate of tiny-TIM-1, a dynamic model of the gastrointestinal tract that includes gastric emptying and digestion product removal by means of dialysis. A possible explanation for the different observations compared to the current study could be that HM contains considerably higher concentrations of (non-AA) NPN than IF (20 vs. 6 % of total N, respectively) [35]. Non-AA NPN consists of small molecules, such as urea, that are easily dialyzed and might therefore contribute to the perceived higher accumulation rate of digestion products, while non-AA NPN does not constitute actual absorbable AA containing fragments.

In the current study, a similar DH increase during the gastric phase was accompanied by a higher intact protein loss for IF than HM, suggesting that per protein molecule more cleavages occurred in HM than in IF (Figure 3 and Figure 5). At the same time, a similar increase in FAA and LMW peptides was observed for HM and IF, but a higher increase in MMW peptides for IF, suggesting that in HM the proportion of gastric digestion products larger than MMW was higher than in IF. One possible explanation could be the smaller average AA chain length of proteins in undigested IF compared to undigested HM. The probability of multiple cleavages within one molecule increases with AA chain length, as does the probability that cleavage products are bigger than 5 kDa (the upper limit of MMW). Indeed, the protein weighted mean molecular weight based on SDS-PAGE analysis was ~28 kDa vs. ~54 kDa for IF and HM, respectively.

A higher intestinal DH increase rate for IF than HM was accompanied by a faster loss of intact protein and an increased release rate of LMW peptides and TP. The DH and TP release at the end of intestinal digestion was similar for IF and HM. Apparently, in the intestinal phase, the remaining peptide bonds in HM contain cleavage sites that are less accessible than those of IF protein. This could be a result of differences in protein composition, processing, and/or matrix between HM and IF.

Intact caseins were more susceptible to gastric hydrolysis than intact whey proteins, in both HM and IF (Figure 6), which is in accordance with previous reports [2,10,11]. During the gastric phase, intact serum albumin in IF was already degraded by 82%, relative to 14% for HM. Particularly intact HM serum albumin, α-lactalbumin, and free secretory component were resistant to intestinal hydrolysis. Intact α-lactalbumin in HM was degraded slower in the intestinal phase than in IF (Figure 6). This could be partly due to the denaturation of α-lactalbumin in IF, which is described to be induced by IF industrial processing [14]. The higher hydrolysis resistance of human milk whey proteins than the cow’s milk counterparts was also previously observed [2].

Although the CM-based IF protein composition was “humanized” with respect to the casein/whey ratio, protein composition still greatly differs between IF and HM. As mentioned, HM contains higher levels of functional whey proteins with functions other than nutritional, such as immunomodulatory, that are different than IF. Interestingly, these are also shown to be relatively resistant to hydrolysis. For example, lactoferrin, secretory immunoglobulin A and lysozyme have been retrieved intact in small amounts (<10% of intake) in the stool of breastfed infants [12]. Although these proteins can also be produced by the infant’s intestine, it has been suggested that the main part has a dietary origin as the stool of breastfed infants contains higher levels than that of IF-fed infants [36]. In line with our observations (Figure 6), other in vitro digestion studies have shown serum albumin, lactoferrin, and immunoglobulins to be the most digestion resistant proteins in (mature) HM after infant in vitro digestion [37]. The higher resistance to hydrolysis of those proteins could be due to post-translational (protective) modifications, such as the high degree of glycosylation observed for human vs. bovine lactoferrin, which hampers hydrolysis by trypsin [38].

Protease inhibitors present in HM, such as α1-antitrypsin and α1-antichymotrypsin [13], may inhibit peptide bond cleavage by the major intestinal proteases. However, concentrations of protease inhibitors in HM show no negative correlation with the overall level of proteolysis during in vitro digestion [37]. Furthermore, the magnitude of their effect on overall protein hydrolysis kinetics is still unknown.

It is well known that the protein composition of HM is dynamic in time, especially during early lactation, the colostrum, and transitional milk periods. After ~30 days of lactation, HM composition becomes less dynamic; this is when the composition is referred to as mature [9]. The HM in the current study was collected 60–251 days postpartum, the obtained results are thus only representative of the mature milk period. The HM sample size in the current study was only 17 donations, the representativeness of average mature HM might therefore also be limited. However, since the mean and median of postpartum days of collection are close (respectively, 157 vs. 167), the collection has a symmetrical distribution and thus represents the whole period.

The manufacturing of IF includes several (heat) processing steps, while HM is typically consumed without any prior treatment. Heat processing has been shown to decrease its susceptibility to hydrolysis due to aggregation and precipitation, as well as due to glycation [14,39], but also to increase whey proteins to hydrolysis due to denaturation [10,40]. Heat-induced protein glycation is unlikely to be responsible for a higher DH increase rate of IF, as this likely has the opposite effect and slows down hydrolysis [14]. Heat processing-induced whey protein denaturation is reported to make whey proteins susceptible to hydrolysis by pepsin, and to induce gastric intact protein loss and DH increase [41]. However, for intestinal digestion, where native whey protein can already be hydrolyzed, an increased rate of intact protein loss is observed at a similar DH increase rate [42]. Whey protein denaturation more likely leads to a change in the hydrolysis mechanism in the intestinal phase by increasing the probability to cleave intact protein over intermediate peptides as described by Adler-Nissen [43]. Therefore, heat-induced protein denaturation is unlikely to be responsible for the observed increased intestinal DH increase rate of IF compared to HM.

The milk matrix in which proteins reside in IF is also different from HM. Typically, in IF the emulsion is comprised of very small lipid droplets (mode diameter <0.5 um) coated with protein, while the HM lipid emulsion structure is comprised of much (>10 times) larger milk fat globules coated with phospholipid membranes. This results in a higher physical association between lipids and proteins in IF compared to HM. However, typical IF emulsions contain only ~24 mg of surface protein per g fat [44], for the currently tested IF this would mean ~13% of the protein is acting as an emulsifier and is thus present on the interface. Moreover, the increased physical association might lessen accessibility for the digestive enzymes. Studies have shown increased hydrolysis of casein when adsorbed at the IF oil–water interface as a result of processing [45], while other studies have shown that homogenization of raw CM leads to slower loss of intact whey proteins during in vitro intestinal digestion [46]. Therefore, the difference in emulsion characteristics between IF and HM does not explain the faster protein hydrolysis.

The abundant proteins, which were found in this study by SDS-PAGE determination, were in line with previous reports [2,11]. However, the HM protein casein to whey ratio (*c*/*w*) of ~27:73 in this study is lower than the expected 40:60 or 50:50 [9]. Contrarily, the *c*/*w* of ~51:49 in IF was higher than the expected 40:60. These differences vs. expectations might be due to individual donor variations in the case of HM, or the specifics of industrial IF production-related selection of (protein) ingredients. We cannot exclude that this skewed protein composition towards casein richness of IF vs. HM contributed to the found faster protein hydrolysis in IF.

Regarding the correlation of in vitro absorbable product release with in vivo postprandial amino acid responses, it needs to be considered that our in vitro model does not include intestinal brush border enzymes, which have been shown to cleave MMW peptides [47,48]. One approach could therefore be to view TP release in vitro to resemble absorbable product release in vivo. It appears that our TP release data correlate with the available in vivo data. Moro et al. reported a 1.18 times higher pp EAA peak of HCM protein-based IF than iso-proteinaceous HM in preterm infants [7]. We congruently observed that in the period where the difference between IF and HM digesta MMW concentrations was maximal, i.e., the first 10–30 min of the intestinal phase, IF digesta contained 1.15–1.17 times more TP. Therefore, TP release assessed in vitro may be a useful proxy for in vivo absorbable product release.

Of the three differences in milk characteristics discussed between HM and IF (i.e., protein composition, heat processing, and product matrix), the first is the most plausible explanation for the observed differences in hydrolysis kinetics and TP release. Hence, to further improve infant formula, and to bring the IF protein digestion rate closer to that of HM, changing the IF protein composition to include more slowly digestible proteins might be an interesting approach to investigate.

## 5. Conclusions

We conclude that the total gastrointestinal digestion product release rate of humanized cow’s milk-based IF is higher than that of HM. The total digestion product release rate assessed in vitro may be useful as a proxy for the in vivo absorbable product release rate. Our results suggest that differences in protein composition are at least partially responsible for the observed differences in release rate. The fact that some HM proteins rely on their intactness to exert their biological function might be a contributing factor to the observed slower hydrolysis in HM. These findings may present an opportunity for further improvement of IF to bring it closer to HM.

## Figures and Tables

**Table 1:** Nutritional composition of the test products ^1^.

g/L	HM	IF ^7^
N	1.72 ± 0.01 ^4^	1.66 ± 0.03
NPN	0.28 ± 0.10 ^4^	0.16 ± 0.02
True protein ^2^	9.00 ± 0.68 ^4^	9.38 ± 0.35
Free AA	0.35 ± 0.02 ^4^	0.02 ± 0.01
Protein equivalent ^3^	9.35 ± 0.69 ^4^	9.40 ± 0.47
Fat	34.00 ± 1.05 ^5^	29.60 ^6^
Carbohydrates	59.50 ± 1.79 ^5^	63.60 ^6^

^1^ Infant milk nutritional composition; N and NPN (non-protein nitrogen, 12% trichloroacetic acid-soluble N) as determined by Dumas, FAA as determined by UPLC. (Means ± sd). ^2^ True protein = ((N-NPN) ×6.25) as recommended by Lonnerdal [9]. ^3^ Protein equivalent (P_eq_) = sum of true protein and free amino acids. ^4^ Data from pooled human milk. ^5^ Weighted means of data obtained using MIRIS human milk analyzer on individual donations. ^6^ Data on pack for 13.60% (*w*/*v*) reconstitution rate converted to used 11.85% (*w*/*v*) reconstitution rate. ^7^ Other components of IF include fructo- and galacto-oligosaccharides.

**Table 2:** Infant milk protein composition as determined by SDS-PAGE and densitometry ^1^.

% of Total Protein Band Intensity	HM	IF
Minor whey proteins	4.50 ± 0.31	-
Lactoferrin	22.54 ± 1.29	-
Serum albumin	10.70 ± 0.31	4.91 ± 0.38
Secretory component of Ig	11.06 ± 2.00	-
α-casein	-	11.61 ± 0.23
β-casein	17.81 ± 1.34	20.35 ± 0.68
κ-casein	8.60 ± 1.04	19.36 ± 0.49
β-lactoglobulin	-	31.18 ± 0.31
α-lactalbumin	24.78 ± 1.15	12.59 ± 0.51

^1^ Means ± SEM (n = 3). IF contains bovine form of the protein, human milk contains human form.

**Table 3:** Curve fitting values of infant milk protein hydrolysis in SIM intestinal phase ^1^.

	HM	IF
**DH**		
k_DH_ (×10^3^) (min^−1^)	26.28 ± 6.27 ^a^	42.92 ± 2.70 ^b^
b_DH_ (%)	7.26 ± 0.28	6.20 ± 0.96
m_DH_ (%)	32.31 ± 2.31	34.61 ± 1.35
R^2^_DH_	0.977 ± 0.001	0.974 ± 0.001
**Free AA**		
k_FAA_ (×10^3^) (min^−1^)	16.44 ± 2.90	26.24 ± 5.23
b_FAA_ (% of P_eq_(0))	6.24 ± 0.23 ^a^	1.76 ± 0.42 ^b^
m_FAA_ (% of P_eq_(0))	25.82 ± 3.21	26.27 ± 1.21
R^2^_FAA_	0.988 ± 0.007	0.982 ± 0.012
**LMW**		
k_LMW_ (×10^3^) (min^−1^)	27.84 ± 1.42 ^a^	72.32 ± 1.22 ^b^
b_LMW_ (% of P_eq_(0))	22.77 ± 1.81 ^a^	16.52 ± 0.78 ^b^
m_LMW_ (% of P_eq_(0))	36.84 ± 1.78	34.76 ± 1.77
R^2^_LMW_	0.988 ± 0.003	0.984 ± 0.005
**Total products**		
k_TP_ (×10^3^) (min^−1^)	31.94 ± 2.04 ^a^	79.65 ± 9.51 ^b^
b_TP_ (% of P_eq_(0))	65.88 ± 2.32	62.98 ± 0.93
m_TP_ (% of P_eq_(0))	48.17 ± 2.39	51.21 ± 0.93
R^2^_TP_	0.981 ± 0.008	0.979 ± 0.005

^1^ Means ± SEM. Means having different letters are significantly different *p* <0.05. k = rate constant, b = value at start of intestinal phase, m = maximal increase in value, R^2^ = coefficient of determination. Suffix DH, FAA, LMW and TP denote respective parameter. AA = amino acids, DH = degree of hydrolysis, LMW = low molecular weight peptides, P_eq_(0) = protein equivalent content of the respective infant milk. TP = total digestion products (sum of free AA, LMW, and MMW). HM = human milk, IF = infant formula.
